# Multidimensional profiles of adolescent functioning: academic, emotional, and relational patterns

**DOI:** 10.3389/fpsyg.2026.1907030

**Published:** 2026-07-30

**Authors:** Millicent Aziku, Xinyi Huang, Denis Djekourmane

**Affiliations:** Faculty of Education, Shaanxi Normal University, Xi’an, China

**Keywords:** academic achievement, adolescence, latent profile analysis, school belonging, whole-child development

## Abstract

**Introduction:**

Adolescence is a critical developmental period during which young people experience significant academic, emotional, and social challenges. While whole-child developmental frameworks advocate integrating academic achievement with psychosocial wellbeing, limited evidence exists on how these dimensions co-occur to form distinct patterns of adolescent functioning. This study identified multidimensional profiles of adolescent functioning across academic, emotional, and relational domains and examined differences in student background characteristics across these profiles.

**Methods:**

Data were drawn from the Programme for International Student Assessment (PISA) 2022 and included 135,348 adolescents (M age = 15.92 years, SD = 0.28) from 15 countries and economies. Latent Profile Analysis (LPA) was conducted using indicators of academic achievement, school belonging, teacher support, emotional wellbeing, and flourishing experiences to identify distinct profiles of adolescent functioning. Differences in student background characteristics across the identified profiles were subsequently examined.

**Results:**

Six distinct profiles of adolescent functioning were identified. One profile exhibited high academic achievement but relatively weak school belonging, lower emotional wellbeing, and reduced flourishing experiences, indicating that strong academic performance does not necessarily coincide with positive psychosocial functioning. In contrast, another profile demonstrated comparatively lower academic achievement alongside strong psychosocial functioning. Significant differences in student background characteristics were observed across the six profiles, suggesting that adolescents’ developmental experiences vary systematically according to their personal and contextual characteristics.

**Discussion:**

The findings support the multidimensional nature of adolescent development and extend the whole-child developmental framework by demonstrating that academic success and psychosocial wellbeing do not always develop in tandem. These results highlight the importance of educational policies and interventions that simultaneously promote academic learning and students’ emotional and relational wellbeing to foster more balanced developmental outcomes.

## 1 Introduction

Adolescence is an important developmental stage characterized by significant cognitive, emotional, social, and academic change. Students in adolescence are presented with particular opportunities and challenges, which are important for their continuous growth and development in terms of academic expectations, relationships with adults and peers, emotional regulations, identity information, and many more (Cantor et al., 2019). The complexities navigated by this cohort of students make it imperative to examine how they differ in their experiences and adaptations in the school environment and learning outcomes. Meanwhile, there is a rise in the advocacy for whole-child development with emphasis on extending adolescent functioning conceptualization beyond academic achievement alone to inculcate their emotional wellbeing, psychological adjustment, social connectedness, and supportive relational experiences within school contexts (Martinsone et al., 2025; Yuen and Wu, 2024). Furthermore, extant research posits that there is a close link between adolescents’ psychosocial experiences within schools and their wellbeing, resilience, engagement, and long-term developmental adjustment (Brown et al., 2025). However, adolescents do not experience these developmental domains uniformly, and growing evidence suggests substantial heterogeneity in how academic, emotional, and relational dimensions co-occur across individuals.

Understanding these multidimensional differences may be particularly important during adolescence, when developmental pathways become increasingly differentiated and educational inequalities may become more pronounced. Although existing research has generated important insights into associations among academic achievement, wellbeing, and school experiences, much of this work has relied on variable-centered approaches that focus on average relationships across populations. Consequently, less is known about how these dimensions combine within adolescents to form distinct developmental profiles. Addressing this gap may provide a more nuanced understanding of adolescent functioning and contribute to whole-child approaches that recognize multiple pathways of adolescent development.

The present study considers constructs such as school belonging, which refers to students’ perceived acceptance, value, and inclusion within their school or classroom environment (Canrinus et al., 2019; Goodenow, 1993; Wang, 2024). In addition, teacher support is included to reflect students’ perceptions of the emotional, instructional, and interpersonal support they receive from their teachers, while emotional wellbeing denotes affective adjustment, indexed by the relative absence of psychological distress and the presence of positive emotional states (Courtney et al., 2023; Tong, 2025). Lastly, flourishing captures a broader constellation of positive psychological functioning, including life satisfaction, resilience, and a sense of meaning and purpose (Waigel and Lemos, 2023). The study treats these constructs as important components of adolescents’ emotional and relational functioning, representing the affective and interpersonal dimension of adolescent development. Analyzing them together with academic achievement forms the multidimensional profiles examined in the present study.

### 1.1 Whole-child development during adolescence

Studies have suggested the adoption of whole-child frameworks that consider individual variability for promoting healthy students’ development and academic success instead of solely focusing on their performance on tests or cognitive achievement (Cantor et al., 2019; Darling-Hammond et al., 2020). This implies that individual functioning should be understood through a comprehensive lens that recognizes the interconnected nature of academic, emotional, social, and psychological development. This sense of connectedness is an integral component of healthy development and educational success. The perspective is particularly important during adolescence, a developmental stage characterized by heightened emotional sensitivity, increasing autonomy, identity exploration, and expanding social expectations (Aslin et al., 2023; Chaku and Davis-Kean, 2024). As adolescents navigate these developmental transitions, schools become critical social contexts that shape not only academic learning but also psychosocial adjustment and developmental wellbeing.

The school environment, especially the social environment, plays a pivotal role in shaping students’ adaptation to academic and social demands. For instance, a positive student-teacher relationship and availability of counseling services that aim at enhancing students’ emotional wellbeing and flourishing are vital for ensuring that students do not only excel academically but also develop healthily (Adomako Gyasi et al., 2025; Zheng, 2022). The interpersonal relationship in the school, if improved, strengthens students’ sense of belonging. Meanwhile, an increased sense of school belonging in itself is an indication of students’ perceptions of being accepted, valued, and included within school communities, whereas teacher support captures students’ perceptions of emotional, academic, and interpersonal support from teachers (Allen et al., 2021; Arslan, 2021; Montecillo et al., 2024). Additionally, emotional wellbeing and flourishing further reflect broader dimensions of positive psychological functioning, including positive emotions, life satisfaction, resilience, and psychological adjustment (Waigel and Lemos, 2023). Evidence from previous scholars demonstrated a consistent association between these developmental experiences and positive educational and psychosocial outcomes, including engagement, motivation, resilience, and academic adaptation during adolescence (Fitrianto, 2025; Klainin-Yobas et al., 2021; Wang, 2024). These perspectives provide an important foundation for examining individual differences in adolescent development within contemporary educational contexts.

### 1.2 Psychosocial and academic dimensions of adolescent functioning

Broad literature on adolescents’ academic functioning has documented that psychosocial experiences within the school and instructional environment play an instrumental role in adolescents’ academic functioning. In particular, teacher support, sense of belonging, wellbeing, and broader indicators of flourishing were expansively explored. For instance, studies posited that the more students reported feeling they belonged in their school environment or being supported by their teachers, the higher their likelihood of active academic engagement, motivation, persistence, and general educational adjustment (Ahn et al., 2021; Allen et al., 2021; Berger et al., 2021; Bureau et al., 2022; Montecillo et al., 2024). When students perceive their school environments as being supportive, it enables them to develop resilience and promote emotional security thereby strengthening their coping mechanisms to navigate developmental and academic challenges that they face at this stage (Azpiazu et al., 2025; Li and Li, 2024; Luo et al., 2023). Similarly, emotional wellbeing and flourishing have been associated with positive developmental outcomes, including life satisfaction, adaptive coping, resilience, and academic adaptation during adolescence (Waigel and Lemos, 2023; Yıldırım and Green, 2024).

Despite these generally positive associations, the existing literature also reveals important inconsistencies in how academic and psychosocial dimensions co-occur among adolescents. Although many studies report positive relationships between wellbeing and academic achievement (DiLeo et al., 2022; Wang et al., 2025), evidence suggests that strong academic performance does not necessarily correspond with positive psychosocial functioning. For example, some adolescents may achieve highly while simultaneously experiencing emotional distress, social disconnection, or reduced wellbeing (Luthar et al., 2020). Conversely, adolescents with comparatively lower academic performance may nonetheless report positive emotional adjustment, supportive relationships, and psychological resilience (Repo et al., 2025; Rudolf, 2024; Tong, 2025). These findings suggest that adolescent functioning is unlikely to follow a single uniform developmental pattern.

Moreover, much of the existing literature has primarily examined isolated relationships among developmental variables using variable-centered approaches. While these studies provide important insights into average associations across populations, they may insufficiently capture the complex ways in which academic, emotional, and relational experiences combine within adolescents. As a result, important multidimensional patterns of adolescent functioning may remain masked. Understanding how these dimensions co-occur may therefore provide a more comprehensive understanding of adolescent development within contemporary educational contexts.

### 1.3 Person-centered perspectives on adolescent heterogeneity

Despite the expansive research on the links among academic achievement, psychosocial wellbeing, and school experiences, much of the existing evidence was based on variable-centered approaches such as regression and structural equation modeling. These approaches primarily estimate average relationships across populations and implicitly assume relative homogeneity in developmental processes among adolescents. However, developmental theory increasingly emphasizes that adolescence is characterized by substantial heterogeneity in how individuals experience and negotiate academic, emotional, and social demands (Chaku and Davis-Kean, 2024). Consequently, average associations may obscure meaningful differences in how developmental dimensions combine across adolescents.

Person-centered approaches offer an important alternative by identifying subgroups of adolescents who exhibit distinct configurations of academic, emotional, and relational functioning. Rather than focusing exclusively on relationships among variables, person-centered methods examine how developmental characteristics co-occur within individuals thereby providing a more holistic understanding of adolescent functioning (Howard and Hoffman, 2018; Spurk et al., 2020). This perspective aligns closely with whole-child developmental frameworks, which conceptualize adolescent adjustment as multidimensional and recognize that adolescents may follow diverse developmental pathways within school contexts (Borgonovi and Pál, 2016; Courtney et al., 2023).

Recent studies employing latent profile and related person-centered approaches have increasingly demonstrated substantial heterogeneity in adolescents’ academic and psychosocial functioning (Doğrusever et al., 2026; Huttunen et al., 2025; Zhang and Jiang, 2023). Nevertheless, limited research has simultaneously integrated academic achievement, school belonging, teacher support, emotional wellbeing, and flourishing within a person-centered framework using large-scale international adolescent data. Addressing this gap may provide a more comprehensive understanding of developmental heterogeneity during adolescence and contribute to contemporary whole-child approaches in educational and developmental research.

## 2 The present study

The present study aims to bridge the literature gap by adopting Latent Profile Analysis (LPA) to examine the heterogeneous patterns of adolescent functioning across academic, emotional, and relational domains. Because LPA is a person-centered analytical method, the current study leverages it to explore distinct profiles based on academic achievement, school belonging, teacher support, emotional wellbeing, and flourishing among adolescents who participated in the 2022 cycle of the Program for International Student Assessment (PISA). By integrating these dimensions within a single analytical framework, the study aimed to provide a more comprehensive understanding of adolescent functioning beyond achievement-centered perspectives.

Beyond identifying multidimensional patterns of adolescent functioning, it is also important to examine whether demographic and educational characteristics are associated with differences across the identified profiles. Previous research has consistently shown that socioeconomic status, gender, immigrant background, and grade repetition are associated with adolescents’ academic achievement, school belonging, emotional wellbeing, and broader educational adjustment (Liu et al., 2025; Yoon et al., 2023). Examining how these characteristics vary across the identified profiles provides a deeper understanding of the populations represented by each profile and helps contextualize the observed patterns of functioning. It may also provide additional insight into how developmental inequalities and educational vulnerabilities are distributed across multidimensional profiles of adolescent functioning.

The study contributes to the literature in several important ways. First, it extends existing research by integrating academic, emotional, and relational dimensions of adolescent functioning within a whole-child developmental framework. Second, the study advances person-centered research on adolescent heterogeneity using a large-scale international dataset. Third, by identifying distinct developmental patterns among adolescents, the study provides a more comprehensive understanding of how academic achievement and psychosocial functioning may align or diverge across individuals during adolescence.

Accordingly, the study addressed the following research questions:

*RQ1*: What latent profiles of adolescent functioning emerge based on academic achievement, school belonging, teacher support, emotional wellbeing, and flourishing?

*RQ2*: How do the identified profiles differ in their multidimensional patterns of academic, emotional, and relational functioning?

*RQ3*: How do demographic and educational characteristics, including socioeconomic status, gender, immigrant background, and grade repetition, vary across the identified profiles?

## 3 Materials and methods

### 3.1 Participants

The analysis for the current study is based on data from PISA 2022. Once every 3 years, the Organization for Economic Cooperation and Development conducts assessment on adolescents who are enrolled in schools or educational institutions at the time of the data collection. Students are administered written tests in reading, mathematics, and science to evaluate their academic competencies (OECD, 2023). To further measure their learning-related experiences, students they presented with questionnaires that collect extensive contextual information regarding their psychological wellbeing, school experiences, and social functioning, making it particularly suitable for examining multidimensional patterns of adolescent development. Some countries and economies administered a wellbeing questionnaire in addition to the general questionnaire administered by all participating countries and economies.

For the current study, the analytical sample comprised 135,348 students from 15 countries and economies that administered the wellbeing questionnaire. Sample ages ranged from 15 to 16 years (*M* = 15.92, SD = 0.28). The country distribution of participants is presented in Table 1. Out of this sample, 49.45% are females, while males represent 50.55%. Native students represent 74.99%, second-generation students represent 11.71, and 13.3% are first-generation students. The average index of economic, social, and cultural status is −0.105 (SD = 1.051). It is important to note that the latent profile analysis aims to examine broad multidimensional patterns of adolescent functioning across the pooled international sample rather than country-specific patterns. Consequently, the identified profiles should be interpreted as global patterns rather than context-specific profiles within individual countries.

**TABLE 1:** Distribution of participants across countries/economies.

Country/ Economy	Frequency (N)	Percentage (%)
Brazil	10,798	7.98
Costa Rica	6,113	4.52
France	6,770	5.00
Hong Kong	5,907	4.36
Hungary	6,198	4.58
Ireland	5,569	4.12
Macao	4,384	3.24
Mexico	6,288	4.65
Netherlands	5,046	3.73
New Zealand	4,682	3.46
Panama	4,544	3.36
Saudi Arabia	6,928	5.12
Slovenia	6,721	4.97
Spain	30,800	22.76
United Arab Emirates	24,600	18.18
Total	135,348	100.00

Frequencies represent the number of participating students included in the analytic sample. Percentages are based on the total sample size (*N* = 135,348).

### 3.2 Ethical consideration

This study is a secondary analysis of publicly available data from the Program for International Student Assessment (PISA) 2022 conducted by the Organization for Economic Co-operation and Development (OECD). No new data were collected, and no participants were directly involved by the authors. Data collection and ethical procedures, including informed consent, voluntary participation, confidentiality, and anonymity, were administered by the OECD and participating countries in accordance with their respective ethical and legal requirements. Therefore, additional institutional ethical approval was not required for this secondary data analysis. Detailed information on the PISA 2022 sampling procedures, data collection, and ethical protocols is available in the OECD PISA 2022 Technical Report.

### 3.3 Instruments

#### 3.3.1 Academic achievement

Students’ scores in reading, mathematics, and science were used to construct their academic achievement, which is consistent with the current study’s multidimensional focus. To measure students’ overall achievement, a composite construct was created using all plausible values across the three PISA domains (PV1READ-PV10READ, PV1MATH-PV10MATH, and PV1SCIE-PV10SCIE), where higher values of academic achievement indicate stronger academic performance. The overall academic achievement values reflect students’ knowledge, competencies, skills, problem-solving, and literacy, among others. To ensure comparability with the psychosocial indicators included in the latent profile analysis, achievement scores were standardized prior to analysis.

#### 3.3.2 School belonging

The current study conceptualizes school belonging as students’ perceived acceptance by peers and others in their school or institutional environment, inclusion, and connectedness to the whole school environment. PISA asked students to report their agreement on six statements related to how they feel in their school [e.g., “I feel like an outsider (or left out of things) at school,” “I feel like I belong at school,” “I feel awkward and out of place in my school,” etc.]. Their responses were rated on a four-point Likert scale ranging from “Strongly agree” to “Strongly disagree.” Because positively worded items were coded such that lower values reflected stronger agreement, relevant items were reverse-coded prior to analysis so that higher values consistently represented stronger school belonging. Standardized factor scores derived from confirmatory factor analyses were used in subsequent analyses.

#### 3.3.3 Teacher support

The study conceptualizes teacher support as students’ rating of emotional and instructional support by their teachers. To measure teacher support, PISA requested students to report their agreement or disagreement on eight statements (e.g., “The teachers at my school are respectful toward me,” “If I came back to visit my school 3 years from now, my teachers would be excited to see me,” “The teachers at my school are mean toward me,” etc.) Responses were rated on a four-point Likert scale ranging from “Strongly disagree” to “Strongly agree.” Negatively worded items were reverse-coded prior to scale construction so that higher scores consistently reflected greater perceived teacher support. Standardized factor scores extracted from the confirmatory factor analysis were used in the latent profile analysis.

#### 3.3.4 Emotional wellbeing

The study measured students’ emotional wellbeing by adopting their reports on the frequency of feeling distress 6 months prior to the assessment. Items included “Feeling depressed,” “Irritability or bad temper,” “Feeling nervous,” “Feeling anxious,” etc. Responses were recorded on a five-point Likert scale ranging from “Rarely or never” to “About every day.” Because higher response values originally reflected greater emotional distress and poorer wellbeing, all items were reverse-coded prior to analysis so that higher values consistently represented more positive emotional wellbeing. The standardized composite score was subsequently included in the latent profile analysis.

#### 3.3.5 Flourishing experiences

Flourishing was operationalized using a standardized composite score derived from six items from the PISA 2022 student questionnaire, which captured adolescents’ positive daily experiences and adaptive functioning. The items assessed dimensions such as accomplishment, positive affect, energy, meaningful engagement, and daily satisfaction. Example of items included are “Did you smile or laugh a lot yesterday?,” “Did you have enough energy to get things done yesterday?,” and “Overall, did you feel that you accomplished something yesterday?” Responses were recorded dichotomously as 1 (Yes) and 2 (No). Relevant items were reverse-coded prior to composite construction so that higher values consistently reflected more positive daily functioning and flourishing experiences. The standardized composite score was subsequently included in the latent profile analysis. Because the items primarily captured diverse daily experiences rather than a narrowly defined latent psychological construct, the measure was operationalized as a standardized composite indicator rather than estimated as a separate latent factor within the confirmatory factor analysis framework.

### 3.4 Data preparation and missing data handling

The data contained some missing values ranging from 0.01% (gender) to 11.61% (emotional wellbeing). The student gender variable in PISA 2022 was assessed using a binary response format (Female/Male), while no additional gender identity categories were available to respondents. The study adopted Multiple Imputation by Chained Equations (MICE) to handle the missing values with the purpose of improving estimation accuracy under the assumption of missing at random (MAR) (Rubin, 1987). This approach prevents listwise deletion of missing or incomplete information. The approach involves generating 20 imputed datasets, and used ordinal logistic regression is for ordinal indicators, logistic regression is for binary variables, multinomial logistic regression is for categorical variables, and linear regression is for continuous variables. To ensure accuracy, all study variables were used for the imputation, including demographic characteristics and academic achievement.

### 3.5 Reliability and measurement validation

The psychometric properties of the psychosocial constructs were evaluated using internal consistency reliability analyses and confirmatory factor analysis (CFA). Cronbach’s alpha coefficients were estimated to assess the internal consistency of the school belonging, teacher support, and emotional wellbeing measures. Relevant items were reverse-coded where necessary to ensure directional consistency across constructs, such that higher scores uniformly reflected more adaptive functioning and positive psychosocial experiences.

Subsequently, CFA was conducted within a structural equation modeling (SEM) framework using maximum likelihood estimation to examine the factorial validity of the psychosocial constructs. The measurement model consisted of three correlated latent dimensions: school belonging, teacher support, and emotional wellbeing. Model adequacy was evaluated using multiple fit indices, including the Comparative Fit Index (CFI), Tucker-Lewis Index (TLI), Root Mean Square Error of Approximation (RMSEA), and Standardized Root Mean Square Residual (SRMR). Consistent with commonly accepted recommendations, CFI and TLI values above 0.90 and RMSEA and SRMR values below 0.08 were considered indicative of acceptable model fit (Hair et al., 2013). Flourishing was operationalized separately using a standardized composite indicator used, which captured adolescents’ positive daily experiences and adaptive functioning.

### 3.6 Analytical procedure

The statistical analysis was conducted in several stages following data preparation in both Stata version 19 and R Studio. First, CFAs were performed to examine the factorial validity of the psychosocial constructs, including school belonging, teacher support, and emotional wellbeing. Standardized factor scores for these constructs were subsequently extracted for use in the latent profile analysis.

Second, LPA was conducted using Gaussian finite mixture modeling to identify distinct configurations of adolescent functioning across academic, emotional, and relational domains. Academic achievement, school belonging, teacher support, emotional wellbeing, and flourishing operated as standardized indicators and were used for the profile construction. Competing profile solutions ranging from 2 to 8 profiles were estimated and compared using multiple statistical and substantive criteria, including the Akaike Information Criterion (AIC), Bayesian Information Criterion (BIC), log-likelihood values, entropy, posterior probability, profile size distribution, and theoretical interpretability (Spurk et al., 2020; Weller et al., 2020). Finally, profile difference analyses were conducted to examine demographic and educational variation across the identified latent profiles. Analysis of variance (ANOVA) was used to assess profile differences in socioeconomic status and academic achievement, whereas chi-square tests were conducted for categorical variables, including gender, immigrant background, and grade repetition. The latent profile analysis was conducted using unweighted pooled data because the primary objective was to identify underlying multidimensional patterns of adolescent functioning rather than estimate nationally representative population parameters.

## 4 Results

### 4.1 Reliability analysis and measurement model fit

The results indicated acceptable internal consistency across school belonging (∝ .82), teacher support (∝ 0.80), and emotional wellbeing (∝ 0.88), which demonstrates that the psychosocial constructs are reliable. Findings from CFA revealed that the model demonstrated acceptable fit to the data *X*^2^ (132) = 82,174.61, *p* < 0.001, CFI = 0.915, TLI = 0.902, RMSEA = 0.068, and SRMR = 0.046). Although the chi-square statistic was statistically significant, this result was expected given the large sample size. Greater emphasis was therefore placed on the approximate fit indices, which collectively supported the adequacy of the measurement structure.

As shown in Table 2, all indicators exhibited acceptable loadings, suggesting that the observed variables adequately represented their respective latent dimensions. The school belonging indicators generally showed substantial loadings ranging from 0.527 to 0.880; loadings for teacher support indicators ranged from 0.602 to 0.914; and loadings for emotional wellbeing items ranged from 0.701 to 0.879, supporting the stability of the three constructs. All factor loadings were statistically significant (*p* < 0.001), providing support for the convergent validity of the latent constructs.

**TABLE 2:** Confirmatory factor analysis results.

Latent construct	Indicator	Standardized factor loading
School belonging	r_ST034Q01TA	0.788
	ST034Q02TA	0.676
	ST034Q03TA	0.689
	r_ST034Q04TA	0.880
	ST034Q05TA	0.527
	r_ST034Q06TA	0.710
Teacher support	r_ST267Q01JA	0.763
	r_ST267Q02JA	0.739
	r_ST267Q03JA	0.852
	r_ST267Q05JA	0.867
	r_ST267Q06JA	0.881
	r_ST267Q07JA	0.914
	ST267Q08JA	0.602
Emotional wellbeing	r_WB154Q04HA	0.701
	r_WB154Q05HA	0.839
	r_WB154Q06HA	0.807
	r_WB154Q07HA	0.879
	r_WB154Q09HA	0.865

All factor loadings were statistically significant at *p* < 0.001. Reverse-coded indicators are denoted by the prefix “r_.”

### 4.2 Latent associations among the constructs

Results in Table 3 revealed positive associations among the latent constructs, such that students who reported stronger teacher support also tended to indicate higher levels of school belonging and better emotional wellbeing. Similarly, greater school belonging was associated with stronger emotional wellbeing, suggesting meaningful interconnections among adolescents’ relational and psychological functioning domains.

**TABLE 3:** Latent covariances among the constructs.

Latent association	Estimate	*p*-value
Belonging ↔ teacher support	0.115	< 0.001
Belonging ↔ emotional wellbeing	0.222	< 0.001
Teacher Support ↔ emotional wellbeing	0.103	< 0.001

Covariances were estimated within the CFA framework using the imputed datasets. All latent associations were statistically significant at *p* < 0.001.

From the analysis, it is observed that the highest covariance emerged between school belonging and emotional wellbeing (estimate = 0.222, *p* < 0.001), indicating that students who experienced stronger social connectedness within the school environment also tended to report more positive emotional functioning. Relatedly, the link between teacher support and school belonging was positive (estimate = 0.115, *p* < 0.001), as well as between teacher support and emotional wellbeing (estimate = 0.103, *p* < 0.001). The finding supports the relational foundations of adolescent psychosocial functioning.

### 4.3 Latent profile model selection

The six-profile solution was selected as the best-fitting and most substantively meaningful model (see Table 4). Although fit continued to improve marginally with additional profiles, model selection was not based on statistical fit alone. Notably, the two-profile solution showed the highest entropy (0.941) of all estimated models; however, entropy reflects classification precision rather than model fit, and the two-profile solution showed poor fit relative to more complex solutions (BIC = 1,786,012) and offered limited substantive resolution of the underlying heterogeneity, and was therefore not selected. The six-profile model showed the lowest BIC (1,737,949) among all estimated solutions, acceptable entropy (0.909), and well-distributed, interpretable profiles ranging from 12.39 to 23.08% of the sample. In contrast, the seven- and eight-profile solutions offered negligible improvement in log-likelihood, lower entropy, and included classes as small as 2.65% and 3.90%, respectively, suggesting overextraction rather than meaningful additional heterogeneity. Classification accuracy for the six-profile solution was high, with average posterior probabilities of profile membership ranging from 0.880 to 0.998 across profiles (see Table 5), supporting the reliability of profile assignment. The decisions regarding profile selection considered statistical indices alongside parsimony, classification quality, and theoretical interpretability, leading to the retention of the six-profile model as the final solution (RQ1).

**TABLE 4:** Fit indices and profile distributions for the latent profile solutions.

Profiles	Log-Likelihood	AIC	BIC	Entropy	Profile distribution (%)
2	−892,763.70	1,785,609	1,786,012	0. 941	65.19, 34.81
3	−889,457.30	1,779,035	1,779,623	0.871	33.49, 43.41, 23.10
4	−882,976.60	1,766,095	1,766,792	0.870	21.43, 30.15, 42.17, 6.25
5	−879,988.20	1,760,152	1,761,016	0.868	30.41, 15.38, 20.92, 27.50, 5.79
6	−868,354.10	1,736,918	1,737,949	0.909	19.15, 14.30, 12.39, 15.95, 23.08, 15.13
7	−868,301.11	1,736,844	1,738,043	0.903	7.09, 14.79, 2.65, 26.76, 10.29, 24.14, 14.28
8	−868,290.01	1,736,858	1,738,225	0.849	3.90, 12.81, 11.45, 8.97, 14.71, 8.17, 27.23, 12.76

AIC, Akaike Information Criterion; BIC, Bayesian Information Criterion. Lower AIC and BIC values indicate improved model fit. For Entropy, higher values indicate better fit.

**TABLE 5:** Classification quality for the selected six-profile solution.

Assigned profile	Profile 1	Profile 2	Profile 3	Profile 4	Profile 5	Profile 6
Profile 1	**0.991**	0.001	0.007	0.001	0.000	0.000
Profile 2	0.001	**0.886**	0.000	0.000	0.112	0.000
Profile 3	0.002	0.000	**0.998**	0.000	0.000	0.000
Profile 4	0.002	0.000	0.000	**0.891**	0.000	0.107
Profile 5	0.001	0.119	0.000	0.000	**0.880**	0.000
Profile 6	0.001	0.000	0.000	0.116	0.000	**0.883**

Values represent the average posterior probabilities of profile membership for individuals assigned to each latent profile. Values on the diagonal (boldface) indicate the average probability of correct classification, whereas off-diagonal values indicate the probability of misclassification. An entropy value of 0.909 indicates excellent classification accuracy.

### 4.4 Profiles of adolescent functioning

Research Question 2 examined the multidimensional profiles of adolescent functioning identified through latent profile analysis. Table 6 summarizes the standardized means and sample distribution for each of the six latent profiles. The standardized means for academic achievement, school belonging, teacher support, emotional wellbeing, and flourishing across the six latent profiles indicate that academic, emotional, and relational domains did not align uniformly across adolescents. Rather than representing a simple continuum from high to low functioning, the profiles reflected qualitatively distinct developmental patterns.

**TABLE 6:** Characteristics of the 6 latent profiles of adolescent functioning.

Profile	Achievement	Belonging	Teacher support	Emotional wellbeing	Flourishing	%
Flourishing and socially connected adolescents	−0.04	0.48	0.33	1.22	1.06	19.15
Academically adaptive but emotionally strained adolescents	0.25	−0.09	0.03	−0.55	0.73	14.30
Globally vulnerable and disengaged adolescents	−0.80	−0.43	−0.40	−0.87	−0.83	12.39
Socially supported but academically vulnerable adolescents	−0.61	1.20	1.09	−0.05	−0.51	15.95
High-achieving but psychosocially disconnected adolescents	0.82	−0.62	−0.44	−0.80	−0.81	23.08
Emotionally positive but relationally disconnected adolescents	−0.06	−0.11	−0.23	0.79	−0.72	15.13

Values represent standardized means (z-scores). Positive values indicate above-average levels relative to the total sample, whereas negative values indicate below-average levels.

Students in the most populated profile (23.08%), are labeled as “High-Achieving but Psychosocially Disconnected Adolescents.” Students in this profile obtained the highest score on academic achievement (*z* = 0.82), but had the lowest score on school belonging (*z* = −0.62) as well as teacher support, revealing relationship challenges faced by this cohort of students. Apart from the Globally Vulnerable and Disengaged Adolescents profile (*z* = −0.87), this profile exhibited the weakest emotional wellbeing (*z* = −0.80). They also reported low flourishing experiences (*z* = −0.81), suggesting that high academic achievement may coexist with substantial psychosocial vulnerability. On the contrary, the “Flourishing and Socially Connected Adolescents” profile (19.15%) exhibited very high emotional wellbeing (*z* = 1.22) and flourishing experiences (*z* = 1.06), alongside elevated belonging (*z* = 0.48) and teacher support (*z* = 0.33), despite near-average academic achievement (*z* = −0.04). These findings indicate that optimal psychosocial functioning does not necessarily correspond to exceptional academic performance.

The “Globally Vulnerable and Disengaged Adolescents” profile (12.39%) demonstrated consistently low scores across all domains, including academic achievement (*z* = −0.80), emotional wellbeing (*z* = −0.87), flourishing experiences (*z* = −0.83), belonging (*z* = −0.43), and teacher support (*z* = −0.40), reflecting a pattern of cumulative disadvantage. By contrast, the “Socially Supported but Academically Vulnerable Adolescents” profile (15.95%) reported the highest levels of belonging (*z* = 1.20) and teacher support (*z* = 1.09) while maintaining below-average academic achievement (*z* = −0.61) and flourishing experiences (*z* = −0.51). This profile suggests that positive relational support alone may not fully offset academic difficulties.

**FIGURE 1:**
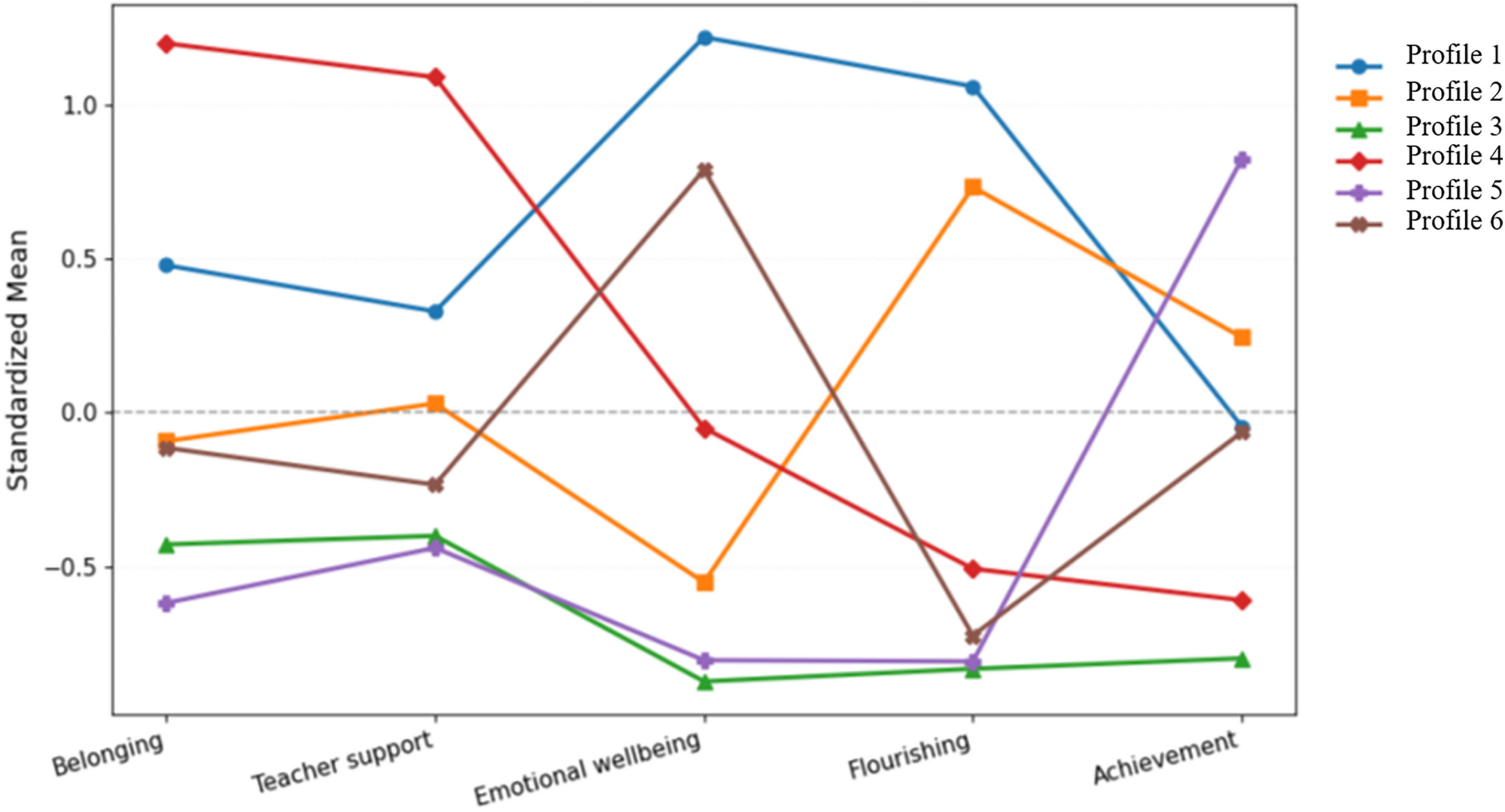
Multidimensional profiles of adolescent functioning. Values represent standardized means (z-scores). Positive values indicate above-average functioning relative to the full sample, whereas negative values indicate below-average functioning.

Two additional profiles reflected more nuanced patterns of functioning. The “Academically Adaptive but Emotionally Strained Adolescents” profile (14.30%) demonstrated above-average achievement (*z* = 0.25) and flourishing experiences (*z* = 0.73) despite low emotional wellbeing (*z* = −0.55) and slightly below-average belonging (*z* = −0.09). Meanwhile, the “Emotionally Positive but Relationally Disconnected Adolescents” profile (15.13%) displayed high emotional wellbeing (*z* = 0.79) despite below-average belonging (*z* = −0.11), teacher support (*z* = −0.23), and flourishing experiences (*z* = −0.72). Collectively, these findings highlight the multidimensional and non-linear nature of adolescent functioning, demonstrating that academic success, emotional wellbeing, and relational connectedness may diverge substantially across adolescents. Figure 1 presents the profile characteristics for easier comparison.

### 4.5 Demographic and educational characteristics across profiles

Table 7 presents the demographic and educational characteristics associated with each latent profile (RQ3). Significant differences emerged across profiles in academic achievement, socioeconomic status, gender distribution, immigrant background, and grade repetition, indicating that the profiles were both psychologically and educationally meaningful.

**TABLE 7:** Demographic and educational characteristics across latent profiles.

Profile	Female (%)	ESCS	Immigrant (%)	Repetition (%)
Flourishing and socially connected adolescents	64.5	−0.12	22.7	16.0
Academically adaptive but emotionally strained adolescents	42.2	−0.09	23.3	13.2
Globally vulnerable and disengaged adolescents	41.1	−0.51	16.9	20.1
Socially supported but academically vulnerable adolescents	39.6	−0.30	19.0	22.3
High-achieving but psychosocially disconnected adolescents	39.0	0.24	22.7	5.4
Emotionally positive but relationally disconnected adolescents	62.9	−0.10	19.6	12.9

The “High-Achieving but Psychosocially Disconnected Adolescents” profile demonstrated the highest socioeconomic status (ESCS = 0.24) and the lowest repetition rate (5.4%), despite reporting comparatively weak belonging, teacher support, emotional wellbeing, and flourishing experiences. This profile also contained a relatively lower proportion of girls (39.0%). In contrast, the “Globally Vulnerable and Disengaged Adolescents” profile exhibited the lowest socioeconomic status (ESCS = −0.51), coupled with elevated repetition rates (20.1%), suggesting the coexistence of academic, psychosocial, and structural disadvantage.

The “Socially Supported but Academically Vulnerable Adolescents” profile displayed the highest repetition rate (22.3%) despite reporting very high belonging and teacher support, further indicating that relational support alone may not fully compensate for academic vulnerability. Conversely, the “Flourishing and Socially Connected Adolescents” profile contained the highest proportion of girls (64.5%) and demonstrated high psychosocial functioning despite near-average academic performance. Similarly, the “Emotionally Positive but Relationally Disconnected Adolescents” profile also showed relatively high female representation (62.9%), suggesting potential gendered differences in psychosocial adaptation patterns.

Although immigrant status differed significantly across profiles, the magnitude of these differences was comparatively modest relative to socioeconomic status and gender. Overall, the findings suggest that the latent profiles were socially stratified and educationally consequential, with distinct configurations of psychosocial functioning associated with varying levels of socioeconomic advantage, academic risk, and demographic composition.

### 4.6 Profile difference analyses

Significant profile differences were observed for academic achievement, socioeconomic status (ESCS), gender, immigrant status, and grade repetition (all *p* < 0.001). The analysis of variance results indicated substantial variation in academic achievement across the profiles, *F*(5, 135,342) = 8989.00, *p* < 0.001, supporting the distinctiveness of the academic patterns characterizing the latent profiles. Similarly, significant differences in socioeconomic status were observed across the profiles, *F*(5, 135,342) = 1176.00, *p* < 0.001, suggesting that adolescents’ developmental profiles were associated with differing levels of socioeconomic advantage and disadvantage.

The chi-square analyses further demonstrated significant demographic variation across the profiles. Significant differences were observed for gender, *X*^2^(10) = 6843.40, *p* < 0.001, immigrant status, *X*^2^(10) = 472.08, *p* < 0.001, and grade repetition, *X*^2^(5) = 2683.20, *p* < 0.001. Collectively, these findings indicate that the identified profiles were meaningfully differentiated not only by psychosocial and academic functioning but also by broader demographic and educational characteristics. In addition, Tukey HSD tests indicated that most pairwise comparisons were statistically significant, with only a small number of non-significant comparisons observed between profiles that exhibited similar scores on individual indicators (see Appendix 1).

## 5 Discussion

Research on adolescent educational development has predominantly focused on academic achievement and how certain emotional, psychological, or relational factors predict it. The current study assumes that there exist different patterns of adolescent functioning across academic, emotional, and relational domains and therefore adopted LPA to explore the multidimensional characteristics of adolescents who participated in PISA 2022. The findings revealed substantial heterogeneity in how academic achievement, school belonging, teacher support, emotional wellbeing, and flourishing experiences co-occurred across adolescents.

### 5.1 Latent profiles of adolescent functioning (RQ1)

The study revealed six profiles, namely, Flourishing and Socially Connected Adolescents, Academically Adaptive but Emotionally Strained Adolescents, Globally Vulnerable and Disengaged Adolescents, Socially Supported but Academically Vulnerable Adolescents, High-Achieving but Psychosocially Disconnected Adolescents, and Emotionally Positive but Relationally Disconnected Adolescents. These findings imply that rather than reflecting a simple continuum from high to low functioning, adolescents may simultaneously experience developmental strengths and vulnerabilities across different domains of functioning. Consistent with prior studies, the results emphasized the importance of whole-child developmental perspectives by demonstrating that adolescent functioning is not a simple linear pattern but a complex non-linear dimension, characterized by diverse developmental configurations (Borgonovi and Pál, 2016; Courtney et al., 2023). In particular, the study suggests that person-centered perspectives should be adopted when analyzing adolescents’ academic development in relation to their psychosocial and relational dimensions.

### 5.2 Multidimensional patterns of academic, emotional, and relational functioning (QR2)

Findings from the study revealed an interesting pattern, especially among students with the profile of “High-Achieving but Psychosocially Disconnected Adolescents.” While students within this category scored relatively high on academic performance, their emotional and relational characteristics such as school belonging, support, emotional wellbeing, and flourishing experiences were relatively low. The findings indicate that some students are academically robust despite their social or emotional sensitivity, which is consistent with developmental research that indicates that high academic pressure could increase emotional distress, social isolation, and psychological problems that, in turn, could negatively affect students’ mental health (Luthar et al., 2020). The findings also contribute to prior work showing that academic competency alone may not be sufficient to capture adolescent wellbeing and adjustment, as a previous study found a positive link between increased study time and academic stress (DiLeo et al., 2022). From a whole-child developmental perspective, adolescents’ academic performance and psychosocial functioning should not be assumed to develop uniformly (Martinsone et al., 2025; Yuen and Wu, 2024). Educational systems that prioritize achievement while neglecting relational and emotional experiences may therefore overlook important developmental vulnerabilities among academically successful students.

In addition, the pattern displayed by the “Flourishing and Socially Connected Adolescents” and the “Emotionally Positive but Relationally Disconnected Adolescents” profiles emphasized developmental assertion indicating that adolescents may achieve positive psychosocial adaptation through multiple pathways rather than through academic success alone. The findings are broadly consistent with research emphasizing the protective role of supportive school relationships, belonging, and emotional adjustment during adolescence (Ahn et al., 2021; Allen et al., 2021; Brown et al., 2025). At the same time, the profiles suggest that positive emotional functioning does not always require exceptionally high academic performance, further challenging narrowly achievement-oriented definitions of adolescent success (Luthar et al., 2020).

The findings additionally revealed a “Globally Vulnerable and Disengaged Adolescents” profile characterized by consistently low academic achievement, weak relational connectedness, poorer emotional wellbeing, and reduced flourishing experiences. This pattern reflects the accumulation of multiple developmental risks and supports ecological and developmental theories emphasizing the interconnected nature of adolescents’ academic, emotional, and contextual experiences (García-Moya et al., 2015; Hennessey et al., 2024). Adolescents within this profile may experience compounding disadvantages across school, emotional, and social domains, potentially placing them at elevated risk for broader maladaptive developmental outcomes. By contrast, the “Socially Supported but Academically Vulnerable Adolescents” profile demonstrated exceptionally high sense of belonging and teacher support despite comparatively low academic achievement and flourishing experiences, demonstrating that relational support alone may not fully compensate for academic difficulties, although supportive school environments may nevertheless function as important protective resources for academically vulnerable adolescents.

### 5.3 Demographic and educational differences across profiles

The results based on demographic characteristics across profiles emphasizes how structural inequalities shape adolescents’ comprehensive development. Evidently, adolescents classified within the more adaptive profiles generally reported comparatively higher socioeconomic status and lower rates of grade repetition, whereas the more vulnerable profiles demonstrated greater socioeconomic disadvantage and elevated repetition rates. These findings are consistent with prior research indicating that socioeconomic resources are closely associated with educational opportunities, psychosocial adjustment, and developmental wellbeing during adolescence (Porter-Stransky et al., 2024; Wang et al., 2023). However, the findings also demonstrated that socioeconomic advantage did not necessarily guarantee positive psychosocial functioning.

Notably, the “High-Achieving but Psychosocially Disconnected Adolescents” profile exhibited the highest socioeconomic status despite comparatively weak school belonging, teacher support, emotional wellbeing, and flourishing. One possible explanation is that socioeconomic advantage provides access to educational resources, learning opportunities, and parental support that facilitate academic success (Addido et al., 2025; Lee and Borgonovi, 2022; Uddin et al., 2024), but it does not necessarily protect adolescents from psychosocial difficulties. Instead, adolescents from more advantaged backgrounds may experience heightened academic expectations, competitive school environments, and pressure to maintain high performance, which can contribute to emotional distress, social disconnection, and reduced wellbeing. This interpretation is consistent with studies showing that adolescents in high-achieving and affluent educational contexts often report elevated levels of stress, anxiety, perfectionistic concerns, and psychological distress despite excellent academic performance (DiLeo et al., 2022; Lin, 2026; Luthar et al., 2020). These findings suggest that academic achievement and psychosocial functioning are related but distinct dimensions of adolescent development, reinforcing the importance of evaluating educational success through a whole-child developmental perspective rather than academic outcomes alone.

The findings additionally revealed notable gender differences across the profiles. Profiles characterized by stronger emotional wellbeing and psychosocial functioning contained substantially higher proportions of girls, whereas academically high-achieving but psychosocially vulnerable profiles contained comparatively fewer girls. This pattern suggests that adolescents may differ in how they experience and respond to academic and psychosocial demands. Previous research indicates that girls generally report stronger interpersonal relationships, greater emotional awareness, and a higher tendency to seek social support, all of which contribute positively to school belonging and psychosocial adjustment (Potter and Yoon, 2023; Yoon et al., 2023). Conversely, boys are often less likely to express emotional difficulties or seek interpersonal support, potentially increasing their vulnerability to psychosocial challenges despite maintaining strong academic performance (Guo et al., 2024). Moreover, although girls often report higher emotional sensitivity, they also tend to benefit more from supportive peer and teacher relationships, which can enhance resilience and overall wellbeing within the school context (Chaplin, 2015). Taken together, these findings suggest that adolescent developmental profiles are shaped not only by academic experiences but also by gendered patterns of emotional regulation, social connectedness, and coping processes. The presence of fewer girls in the academically high-achieving yet psychosocially vulnerable profile further implies that academic success alone may not adequately reflect adolescents’ developmental adjustment, reinforcing the importance of adopting a whole-child perspective that considers both academic and psychosocial dimensions simultaneously.

### 5.4 Practical and theoretical implications

Overall, the current study has some implications for stakeholders in education as they endeavor to promote students’ development. The findings suggest test scores should not be used as the sole metrics for measuring educational success, especially among adolescents who are at a critical developmental stage. This implies that advocacy for quality education should emphasize whole-child education to account for the comprehensive development of students. The identification of adolescents who achieved highly despite comparatively poor psychosocial functioning suggests that achievement-focused educational models may undermine key emotional and relational needs of adolescents. Therefore, educational policies and interventions should promote inclusive school climates, strengthen teacher-student relationships, and support adolescent wellbeing alongside academic learning.

Importantly, the findings also highlight the need to recognize the diversity of adolescents’ developmental experiences. The six latent profiles demonstrate that students do not follow a single pathway of development; rather, they exhibit distinct combinations of academic strengths and psychosocial needs. Consequently, schools should move beyond one-size-fits-all approaches and adopt differentiated support systems that respond to the specific needs of different groups of students. For example, academically successful students may benefit from interventions that strengthen emotional wellbeing and school belonging, whereas academically vulnerable students may require both academic assistance and sustained relational support. Likewise, students experiencing multiple forms of disadvantage may require coordinated academic, psychological, and social interventions. Recognizing this developmental heterogeneity enables educators to design more targeted, equitable, and responsive practices that better support the diverse needs of adolescents within whole-child educational frameworks.

In theory, the findings support whole-child developmental models by showing that academic achievement, emotional wellbeing, and relational functioning are not necessarily aligned in a consistent way across adolescents. Adolescent functioning, instead, seems multifaceted and characterized by different developmental pathways. These findings support person-centered approaches as useful tools for studying variability in adolescence beyond average population-level associations. In particular, the multidimensionality of the profiles shows that no single measure alone may be sufficient to describe adolescent adjustment. Future educational and developmental paradigms may benefit from considering the complexity and variability of adolescent functioning rather than relying solely on academic achievement as the key indicator of successful development.

### 5.5 Limitations and future directions

The study has some limitations that should be considered when interpreting the findings of the present study. First, the study relied on secondary data from PISA 2022, which limited the inclusion of potentially important contextual and developmental variables relevant to whole-child functioning. Factors such as family relationships, peer dynamics, community environments, and clinical indicators of mental health were not comprehensively captured within the dataset. Future research may therefore benefit from the use of primary or mixed-method data collection approaches that allow for a broader and more contextually grounded examination of adolescent development. Second, although PISA provides one of the largest internationally comparative datasets on adolescents, participating countries are disproportionately concentrated among relatively economically developed and educationally established systems. Consequently, the findings may not fully reflect the experiences of adolescents in more economically disadvantaged or educationally under-resourced contexts, where multidimensional vulnerability may manifest differently or more severely. Future studies should therefore examine whether similar developmental profiles emerge within lower-income and educationally marginalized settings. Third, the cross-sectional nature of the data limits causal interpretation of the observed profiles and associations. Adolescent functioning is dynamic and may change substantially over time as students encounter shifting academic, emotional, and relational experiences. Longitudinal person-centered research would therefore provide important insight into the stability, developmental transitions, and long-term implications of different adolescent functioning profiles. Furthermore, relational and emotional variables are based on self-reports, which may be subjected to reporting bias or social desirability. Future studies should consider the use of observational data. The latent profiles were derived from pooled international data, which may restrict potential cultural and contextual variation in adolescent functioning across countries and educational systems. Although the profiles reflected broad global patterns, the meaning and developmental significance of specific psychosocial experiences may vary across sociocultural contexts. Future research should therefore examine the extent to which similar multidimensional profiles replicate across specific countries, regions, and cultural settings. The profile comparisons were conducted using assigned latent profile membership. Although this classify-and-analyze approach does not account for classification uncertainty, entropy was high and average posterior probabilities indicated good classification quality. Nevertheless, the findings should be interpreted with this limitation in mind.

## 6 Conclusion

The present study explored heterogeneity in academic, emotional, and relational constructs among adolescents, based on the PISA 2022 dataset. Latent profile analysis identified six profiles of adolescents with different levels of academic achievement, school belonging, teacher support, emotional wellbeing and flourishing experiences. The findings emphasize the relevance of whole-child development. Adolescent development cannot be adequately understood from any single perspective. Crucially, the profiles suggested that academic success may co-occur with psychosocial vulnerability and that positive emotional and relational functioning may occur despite relatively lower academic attainment. Such findings are consistent with whole-child developmental perspectives that assume adolescent functioning to be multidimensional and sensitive to heterogeneous developmental trajectories (Cantor et al., 2019; Darling-Hammond et al., 2020). This research adds to the literature by bringing together academic, emotional, and relational aspects within a person-centered approach.

## Data Availability

Publicly available datasets were analyzed in this study. This data can be found at: https://www.oecd.org/en/data/datasets/pisa-2022-database.html.

## Appendix 1

**TABLE A1:** Tukey HSD *post hoc* comparisons of the six latent profiles.

Outcome variable	Non-significant pairwise comparison(s)	Adjusted *p*
Academic achievement	Profile 2 vs. Profile 4	0.997
	Profile 5 vs. Profile 6	0.167
School belonging	Profile 2 vs. Profile 5	0.729
	Profile 4 vs. Profile 6	0.824
Teacher support	Profile 2 vs. Profile 4	1.000
	Profile 2 vs. Profile 5	0.642
	Profile 2 vs. Profile 6	0.659
	Profile 4 vs. Profile 5	0.651
Emotional wellbeing	Profile 2 vs. Profile 4	1.000
	Profile 5 vs. Profile 6	0.999
Flourishing	Profile 1 vs. Profile 2	0.918
	Profile 1 vs. Profile 4	1.000
	Profile 2 vs. Profile 4	0.611

One-way analyses of variance indicated significant overall differences among the six latent profiles for academic achievement, school belonging, teacher support, emotional wellbeing, and flourishing (all *p* < 0.001). Tukey’s honestly significant difference (HSD) *post-hoc* tests showed that all remaining pairwise comparisons not listed in this table were statistically significant (adjusted *p* < 0.05).
